# Antioxidant Defences and Redox Homeostasis in Animals

**DOI:** 10.3390/antiox11051012

**Published:** 2022-05-20

**Authors:** Peter F. Surai, Katie Earle-Payne

**Affiliations:** 1 Vitagene and Health Research Centre, Bristol BS4 2RS, UK; 2 Department of Microbiology and Biochemistry, Faculty of Veterinary Medicine, Trakia University, 6000 Stara Zagora, Bulgaria; 3 Department of Animal Nutrition, Faculty of Agricultural and Environmental Sciences, Szent Istvan University, H-2103 Gödöllo, Hungary; 4 NHS Greater Glasgow and Clyde, Renfrewshire Health and Social Care Centre, 10 Ferry Road, Renfrew PA4 8RU, UK; katie.earle-payne@ggc.scot.nhs.uk

For many years reactive oxygen species (ROS) production in biological systems has been considered to be detrimental. However, a pleasant face of ROS has received recently tremendous attention. Indeed, it has been proven that ROS participate in cell signalling, transcription factors regulation and vitagene activation to maintain optimal cellular redox balance and to provide an effective stress adaptation [1]. In fact, redox biology is a very rapidly developing area of modern biological sciences, and regulatory roles of redox homeostasis maintenance in health and development of various diseases has become a hot topic of research worldwide. It is important to emphasize that most research in this exciting area was related to medical sciences in an attempt to develop new approaches and therapeutics for prophylactic and/or treatment of a range of diseases where ROS overproduction and oxidative stress are proven to play detrimental roles [2]. However, it is well accepted that commercial animal/poultry production is associated with a range of stresses, including physiological (extremely fast growth rate, high egg production or meat yield, etc.), environmental (heat stress or dust), technological (chicken placement, transfer from rearing to breeding houses, pig or calf weaning, etc.), nutritional (mycotoxins or oxidised fat in the feed) and internal (vaccination, pathogen challenge, etc.) stresses. Notably, many of the aforementioned stresses cannot be avoided since commercial technologies of egg, meat and milk production are cost-sensitive. It is generally accepted that at the molecular level major aforementioned stresses are associated with ROS overproduction, oxidative stress and redox balance disturbances leading to decreased productive and reproductive performance of poultry/farm animals and economical losses [1]. Physiological roles of redox homeostasis maintenance in poultry and farm animals, have received limited attention and are poorly characterized. However, for the last five years, this topic was a centre of attention for a range of publications covering some related aspects.

Therefore, a Special Issue “Antioxidant Defences and Redox Homeostasis in Animals” includes eight experimental and four review papers devoted to the subject. Among commercially-relevant stresses causing disturbances of the redox status in poultry, mycotoxins and heat stress received major attention in this Special Issue. Indeed, it is well accepted that mycotoxins in the poultry/animal feed are unavoidable nutritional stresses compromising AO defence networks, causing oxidative stress and detrimentally affecting immunity and gut health [3]. It should be mentioned that fusarium mycotoxins cause major problems in poultry and farm animal industries. In particular, it was shown that after a short-term (three-day) in vivo feeding of laying hens with fumonisin B_1_ (FB1; at 40 mg/kg feed) significantly decreased the reduced glutathione (GSH) content and the activity of glutathione peroxidase (GPx) in the liver in comparison to the control group were obseved [4]. However, DON (10 mg/kg) or T-2 toxin (0.5 mg/kg) did not affect those antioxidants. Interestingly, on day three, DON significantly decreased MDA in the liver while other mycotoxins did not affect lipid peroxidation. Furthermore, T-2 toxin (at days one and three) and DON (at day one) were reported to upregulate the expression of the *GPX4* gene, while there was no effect of mycotoxins on the expression of the gene at day two. Glutathione reductase (GR) activity was not affected by mycotoxins, while at day one, DON and FB1 decreased glutathione synthase (GSS) activity and at day two, FB1 significantly increased GSS activity. The authors concluded that the *Fusarium* mycotoxins (T2 toxin, DON and FB1) investigated individually differently affected the antioxidant defence system leading to low-level oxidative stress at the doses studied [4]. In another mycotoxin-related paper, the mechanism of *Hedyotis diffusa* (HD) in mediating the detoxification of aflatoxin B_1_ (AFB_1_)-induced hepatic injury in chicks was investigated [5]. In this experiment, the broiler chicks were fed either a control diet (Control), Control plus AFB_1_ (0.5 mg/kg_)_, or Control plus AFB_1_ (0.5 mg/kg) with HD (at 500 or 1000 mg/kg) for two weeks. Detrimental effects of AFB_1_ were evidenced as liver injury and significantly decreased body weight gain, feed intake and feed conversion ratio. Swelling, necrosis, and severe vacuolar degeneration in chicks’ livers were observed to be induced by AFB_1_. Dietary HD supplementation at the two doses was reported to significantly upregulate antioxidant defences (NRF2, NAD(P)H: quinone oxidoreductase-1, heme oxygenase-1, glutathione cysteine ligase catalytic subunit, and glutathione-S transferase A2 and A3) in livers in comparison to the AFB_1_ group. The authors concluded that protective effects of HD against AFB_1_-induced hepatotoxicity were associated with the activation of NRF2/ARE signaling [5].

Next three papers of this Special Issue deal with heat stress in chickens. The effects of chronic heat stress (8 h at 34 °C) on carcass traits, muscle oxidative stability, muscle fatty acids and amino acid profiles in 100-day-old male Ross broiler chickens were investigated [6]. It was shown that HS compromised AO defences as evidenced by significantly decreased SOD and catalase activity in heart tissues. Furthermore, chronic thermal stress was responsible for the reduced levels of PUFAs and essential AA in broiler chickens and decreased carcass yield and reduced oxidative stability of breast muscles. Interestingly, the oxidative stability of thigh muscles was not affected by the HS conditions [6]. The impacts of dietary curcumin supplementation (100 mg/kg) on muscle oxidative stability and energy metabolism in heat-stressed (HS, 8 h at 34 °C, daily, 22–42 days) broilers were investigated [7]. The authors showed that the dietary curcumin supplementation of HS broilers significantly decreased levels of MDA in the breast and thigh muscles and significantly improved the levels of ATP and CoQ10 in liver tissues. Furthermore, curcumin supplementation was reported to significantly increase the breast yield, reduced the percentage of abdominal fat, significantly improved the levels of monounsaturated fatty acids (MUFAs) and polyunsaturated fatty acids (PUFAs) in breast and thigh muscles [7]. Protective effects of organic Se in the form of OH-SeMet and Se-Yeast (SY) in comparison to traditional sodium selenite (SS) at 0.3 mg/kg in broilers exposed to environmental stress (a high stocking density and heat stress condition for six weeks) were studied [8]. It was demonstrated that OH-SeMet was able to improve the FCR and Se concentration in the tissues (liver, pectoral muscle and jejunum) in comparison to SS and SY. Both organic Se sources (SY and OH-SeMet) were responsible for reduction in the serum cortisol, T3, IL-6, IgA, IgM and LPS in comparison to SS. Similarly, when compared to SS, both SY and OH-SeMet were found to improve the intestinal morphology and increased the T-AOC, TXRND, SELENON and OCCLUDIN activities but decreased CLAUDIN2 in the jejunum. Of note, OH-SeMet further improved these values in comparison to SY. Importantly, SY and OH-SeMet both increased SELENOS and TXNRD2 expression in the muscles compared with the inorganic Se source (SS). In addition, OH-SeMet further raised T-AOC, GPX4, SELENOP, SELENOW and TXNRD1, and reduced MDA and protein carbonyl in the muscles than SS and SY. These observations allowed the authors to conclude that OH-SeMet showed a better ability to maintain the redox and immune status as well as the performance of broilers under a high stocking density and heat stress challenge than SS or SY [8].

Among different stress models used to study protective effects of various antioxidants, poultry semen can be considered as a valuable model due to high levels of polyunsaturated fatty acids in sperm membranes, a range of antioxidant defence mechanisms and easy techniques to add antioxidants to study important semen parameters reflecting membrane integrity and cell viability. Therefore, in a review paper the antioxidant system of avian semen was briefly characterised and the recent knowledge regarding progress in extender supplementation using antioxidants and other compounds to improve avian semen quality and fertility was summarised. Among compounds possessing AO activity, vitamin E, GSH, N-acetyl-L-cysteine, L-carnitine, taurine, coenzyme Q10, lycopene, alpha-lipoic acid, ergothioneine, melatonin, quercetin, as well as AO enzymes SOD and Catalase were tested as diluent additives. A range of oxidative stress-related parameters (AO enzymes, total AOA, lipid peroxidation, mitochondrial integrity, etc.) in diluted or cryopreserved and defrosted avian semen were shown to be improved by the AO additives. Some of them were able to improve fertilizing ability of spermatozoa as well. The authors suggested that nanotechnology and encapsulation could help in delivery of fat-soluble substances to the semen and improve their protective effects [9]. Another review paper devoted to redox homeostasis in poultry described in detail regulatory roles of NF-κB. In our previous review paper transcription factor Nrf2 was described as a master regulator of antioxidant defences in avian species via activation of various vitagenes and other protective molecules to maintain redox homeostasis in cells/tissues [10]. Recently, it has been shown that Nrf2 is closely related to another transcription factor, namely, NF-κB, responsible for control of inflammation. Therefore, in a review published in this Special Issue [11] a current view on NF-κB functioning in poultry with a specific emphasis to its nutritional modulation under various stress conditions was presented. In particular, it has been clearly shown that, in many stress conditions in poultry, NF-κB activation is responsible for increased synthesis of proinflammatory cytokines leading to systemic inflammation, which is a major problem for poultry industry. At the same time, there are a range of nutrients/supplements (carnitine, taurine, silymarin, selenium, etc.) that can downregulate NF-κB and decrease the negative consequences of stress-related disturbances in redox homeostasis. In general, Nrf2-vitagene–NF-κB interactions in relation to redox balance homeostasis maintenance, immunity, and gut health in poultry and animal production await further research [11].

A potential of dietary organic acids in regulation of redox homeostasis, immunity, and microflora in intestines of weaned piglets was described [12]. It was shown that organic acid blends OA1, containing formic acid (31.0%), ammonium formate (23.0%), and acetic acid (8.3%) and OA2 containing formic acid (13.0%), ammonium formate (19.0%), acetic acid (10.0%), propionic acid (13.0%), sorbic acid (0.5%), lactic acid (1.0%), and citric acid (0.5%) improved the AO defence system as evidenced by increased serum CAT and SOD activities and T-AOC and decreased MDA concentration. The same blends improved gut health by increasing the jejunal expression of host defence peptides (*PBD1*, *PBD2*, *NPG1,* and *NPG3*) and tight junction genes (claudin-1) and decreased expression of proinflammatory cytokines (*IL-1β* and *IL-2*). This was associated with improved growth performance, decreased piglets’ diarrhoea rate and improved small intestinal morphology [12]. The effect of a diet supplemented with fresh amla fruit (FAF) as a natural feed additive on milk antioxidant capacity, milk fatty acid (FA) composition and blood metabolic parameters in lactating dairy cows were investigated [13]. The five main metabolites in FAF were shown to be phenolic acids (22%), flavonoids (20%), lipids (20%), amino acids and derivatives (9%), and tannins (7%). The FAF dietary supplementation (400 g/day) was found to increase the antioxidant capacity biomarkers in the blood, including SOD, improved ferric reducing-antioxidant power (FRAP) and total antioxidant capacity (TAC) in milk compared to controls. Furthermore, FAF changed fatty acid profile of milk by reducing proportions of saturated fatty acid and the omega-6/omega-3 ratio and increasing proportions of PUFAs in a dose-dependent manner [13]. In addition, a review paper on oxidative stress in dairy cows clearly showed that lipid peroxidation (as MDA) and SOD activities increased in high concentrate-fed cows accompanied by a reduction of total antioxidant capacity (T-AOC), GPx and catalase activity. Moreover, albumin and paraoxonase concentrations were shown to be inversely related to oxidative stress and high concentrate diets were shown to be pro-inflammatory as evidenced by increased expression of MAPK pro-inflammatory genes simultaneously with reduction in the expression of antioxidant genes and proteins in mammary epithelial tissues. A high-grain or high concentrate diet was shown to down regulate Nrf2 with simultaneous upregulation of NF-κB. The authors have shown that amino-acids, vitamins, trace elements, and plant extracts could be promising compounds to enhance immune functions and repairing damaged cells exposed to oxidative stress [14].

It should be noted that diets can regulate AO defences and redox status also in fish. In fact, supplemental microalgal DHA was shown to affect redox status of juvenile rainbow [15]. In experiments, two astaxanthin (AST) sources (at 80 mg/kg, synthetic, or SA, vs. microalgal, or AA) and three levels (0%, 50%, and 100%) of fish oil substitution with DHA-rich Aurantiochytrium microalgal meal were tested. It was shown that major redox enzymes including GR, GPX, GST, and SOD in the muscle and liver of trout were affected by the diets tested. In fact, gene expressions and activities of major antioxidant enzymes were shown to be suppressed by FO substitution with DHA-rich microalgae in the liver and muscle of rainbow trout, while dietary source and (or) concentration of AST did not affect functional expression of antioxidant enzymes in the liver or muscle of trout [15].

Among many different antioxidants in the body, taurine (Tau), a sulphur-containing non-proteinogenic β-amino acid, is believed to be an important natural modulator of the antioxidant defence networks and redox homeostasis. A review paper in this Special Issue describes AO properties of Tau in detail [16]. It is well known that Tau is synthesised in most mammals and birds and its requirement is met by both internal synthesis and food/feed supply. Tau’s antioxidant protective action is of great importance under various stress conditions associated with commercial animal and poultry production. It might be assumed that in stress conditions, Tau synthesis could be compromised while requirement would be increased, a situation when optimal Tau dietary supply would play an important protective role. Analysis of current literature clearly indicates that the direct antioxidant effect of Tau due to scavenging free radicals is limited, while the stabilising effects of Tau on mitochondria deserve more attention. Antioxidant protective effects of Tau were clearly demonstrated in various in vitro and in vivo toxicological models. It seems likely that the membrane-stabilizing effects, inhibiting effects on ROS-producing enzymes, as well as the indirect AO effects of Tau via redox balance maintenance associated with the modulation of various transcription factors (e.g., Nrf2 and NF-κB) and vitagenes are the main molecular mechanisms of the Tau protective action under various stress conditions [16]. A growing body of evidence related to various stress models in poultry (e.g., heat stress, immunological challenge, increased stocking density, and presence of toxicants in the diet) clearly showed protective effects of dietary Tau associated with improved AO defences and maintenance of the redox homeostasis [17].

Therefore, main emphasis in this Special Issue was given to redox balance maintenance in poultry (eight papers), with two papers related to dairy and one paper deals with pigs and another paper is devoted to fish. It has been illustrated that redox signalling is integrated with main homeostatic mechanisms [18] and associated with the vitagene network and various transcription factors to maintain redox homeostasis [1]. Poultry and farm animals manage stress via adopting various mechanisms generally termed ‘stress response’ (SR) based on induction of a range of genes regulating synthesis of various cyto-protective molecules. There are at least eight stress response pathways participating in stress sensing and development of the adequate response via maintenance/re-establishment of the redox balance (Figure 1).

As mentioned above, three papers in this Special Issue deal with heat stress and two papers are devoted to mycotoxins in poultry. It is known that stresses in poultry production caused by high temperature and mycotoxins, can activate heat shock response [20]. It is also known that various pathogens, damaged macromolecules, allergens and various chemicals can upregulate inflammatory stress response (ISR) associated with activation/translocation to nucleus of NF-κB and synthesis of various pro-inflammatory cytokines [19] as it was described in detail in the review published in this Special Issue [11]. Nutritional disbalance/inadequacy and protein oxidation can activate nutritional stress response (NSR) associated with AMPK induction and autophagosome formation with following lysosomal digestion of damaged mitochondria [19]. This, together with unfolded protein response, could be relevant to the paper describing benefit of organic Se in poultry nutrition in this Special Issue [8]. Indeed, the benefit of organic Se is based on the ability of SeMet to be non-specifically incorporated into proteins, including muscle proteins, building Se reserves in the body [21] which can be used in stress conditions when Se consumption decreased as a result of reduction in feed consumption. However, its requirement increased due to a need for additional selenoprotein synthesis. The molecular mechanisms regulating conversion of SeMet accumulated in tissues, for example in muscles, to H2Se and further to synthesis of SeCys and its incorporation into newly synthesised selenoproteins are not clear at present but it seems likely that changes in redox status of the cells/tissues and activation of proteasomal protein degradation could be involved [21]. Indeed, ATP- and ubiquitin-independent proteolysis by the 20S proteasome is shown to be involved in the selective degradation of oxidised proteins and the 20S proteasome demonstrated an increased proteolytic activity toward oxidised polypeptides. Indeed, a 30% decreased activity of the chymotrypsin-like activity of proteasome in cells overexpressing GPx1 was reported [22]. In the same paper it was described that exposure of HeLa cells to antioxidants was associated with the reduced proteasome 20S chymotrypsin-like activity. It seems likely that Se status could control the proteasome activity and this could be considered as a feedback mechanism of recognition of SeMet as a source of Se for selenoprotein synthesis. Therefore, under stress conditions some amino acids of muscle proteins could be oxidised, and this would trigger an increase in proteasome activity to degrade such proteins leading to a release of SeMet which could be available for additional selenoprotein synthesis. When redox balance/homeostasis is restored, increased GPx activity would decrease proteasome activity and protein degradation [21]. Finally, oxidative stress response is associated with Nrf2 and vitagene activation and additional synthesis of a range protective molecules [10] and this mechanism is relevant to the data presented in the paper by Zhao et al. [5] showing the protective effect of *Hedyotis diffusa* against AFB_1_-induced hepatotoxicity in chickens. Furthermore, activation of oxidative stress response is an important mechanism of the taurine AO action [16] and protection against oxidative stress in cows [14] described in this Special Issue.

In conclusion, 12 papers published in this Special Issue described various aspects of antioxidant system and redox balance regulation and possibilities of their modulation by nutritional means in poultry, pigs, dairy and fish. Roles of redox balance/homeostasis in mammalian oocyte and embryo development [23], avian embryo development [24], immunity [25], inflammation [26], gut health [27], meat quality maintenance [28], and prevention of wooden breast myopathy in broiler chickens [29] are of paramount importance. A better understanding of the oxidative stress mechanisms and roles of the redox homeostasis in prevention/treatment of commercially relevant stresses in poultry and farm animal nutrition allows to develop effective feed additives as mitigators of the negative consequences of stresses [30]. In addition to the nutritional antioxidant-modulating supplements described in the aforementioned papers, including organic Se [8], curcumin [7], organic acids [12], Fresh *Phyllanthus emblica* Fruit [13], and *Hedyotis diffusa* [5], it seems likely that such nutrients as silymarin [31], taurine [17,32] and carnitine [33] deserve more attention. Indeed, more research in this exciting area is expected in the near future [34,35].

## Figures and Tables

**Figure 1:**
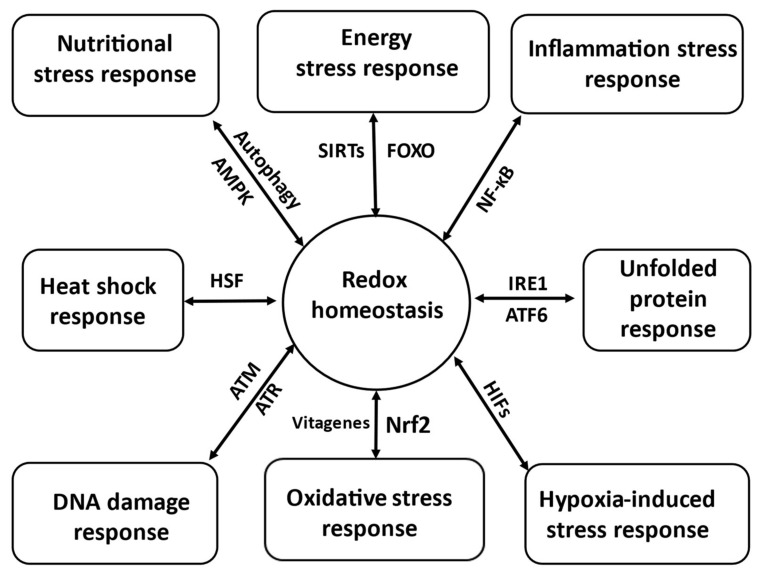
Hypothetical redox homeostasis and stress-response interaction scheme (adapted from [1,10,11,18,19]. AMPK, AMP-activated protein kinase; ATF6, activating transcription factor 6; ATM, ataxia-telangiectasia-mutated; ATR, ataxia-telangiectasia and Rad 3-related; FOXO, forkhead box protein; HIF, hypoxia inducible factor; HSF, heat shock factor; IRE1, inositol-requiring enzyme 1; NF-κβ, nuclear factor kappa-light-chain-enhancer of activated B cells; Nrf2, nuclear factor erythroid-2 related factor 2; SIRTs, sirtuins.

## Abbreviations

AA	amino acid
AFB1	aflatoxin B1
AMPK	AMP-activated protein kinase
AO	antioxidant
ATF6	activating transcription factor 6
ATM	ataxia-telangiectasia-mutated
ATR	ataxia-telangiectasia and Rad 3-related
CAT	catalase
DON	deoxynivalenol
FA	fatty acids
FB1	fumonisin B_1_
FO	fish oil
FOXO	forkhead box protein
FRAP	ferric reducing-antioxidant power
GPx	glutathione peroxidase
GR	glutathione reductase
GSH	reduced glutathione
GSS	glutathione synthase
GST	glutathione S-transferase
HD	*Hedyotis diffusa*
HIF	hypoxia inducible factor
HS	heat stress
HSF	heat shock factor
IRE1	inositol-requiring enzyme 1
LPS	lipopolysaccharide
MAPK	mitogen-activated protein kinase
MDA	malondialdehyde
NF-κβ	nuclear factor kappa-light-chain-enhancer of activated B cells
Nrf2	nuclear factor erythroid-2 related factor 2
PUFAs	polyunsaturated fatty acids
ROS	reactive oxygen species
SeMet	selenomethionine
SIRTs	sirtuins.
SOD	superoxide dismutase
SR	stress response
SS	sodium selenite
SY	Se-Yeast
TAC	total antioxidant capacity
T-AOC	total antioxidant capacit
Tau	taurine
TXRND	thioredoxin reductase
